# Effect of Mechanical and Thermal Aging on the Retention Stability of Attachment Systems for Mandibular Implant Overdentures

**DOI:** 10.3390/jfb17100523

**Published:** 2026-10-16

**Authors:** Mustafa Yıldırım, Murat Alkurt

**Affiliations:** 1 Department of Prosthodontics, Faculty of Dentistry, Niğde Ömer Halisdemir University, 51240 Niğde, Turkey; 2 Department of Prosthodontics, Faculty of Dentistry, Recep Tayyip Erdoğan University, 53000 Rize, Turkey

**Keywords:** implant overdenture, attachment systems, retention, mechanical aging, thermal cycling, dental biomaterials

## Abstract

Background/Objectives: This descriptive pilot in vitro study characterized changes in retentive force in four attachment-specific overdenture specimens for a four-implant mandibular overdenture model following mechanical and thermal aging and across different force-application geometries. Methods: Four parallel implants were placed in the interforaminal region of an edentulous polyurethane mandibular model. One attachment-specific overdenture specimen was fabricated for each condition: distal extension bar, ball/O-ring, Locator R-Tx, and TC-Snap with Retention.sil 600. Retentive force was measured at four unilateral and three bilateral dislodgement points at baseline, after 540 and 1080 insertion–removal cycles, and after 5000 and 10,000 thermal cycles. Ten consecutive pull-out measurements at each test point and measurement interval were obtained from the same specimen and treated as technical replicates rather than independent experimental units; the data were summarized descriptively. Results: After 1080 insertion–removal cycles, proportional retention losses were 32.50% for the distal extension bar specimen, 17.18% for the ball/O-ring specimen, 16.06% for the Locator R-Tx specimen, and 16.91% for the TC-Snap specimen. After 10,000 thermal cycles, the corresponding losses were 47.42%, 30.11%, 30.78%, and 28.02%, respectively. The tested distal extension bar specimen maintained the highest absolute retentive-force values but showed the greatest proportional loss. Point-specific and unilateral–bilateral retention patterns varied across the four tested specimens. Conclusions: Within the limitations of this descriptive pilot design, absolute retentive force and proportional retention loss represented distinct aspects of the retention behavior observed in the tested specimens, while force-application geometry was also associated with different descriptive retention patterns.

## 1. Introduction

Severe resorption of the mandibular residual ridge remains a major challenge in the prosthetic treatment of edentulous patients because it can compromise the retention and stability of conventional mandibular complete dentures and negatively affect oral function and patient satisfaction [1,2]. Mandibular implant-supported overdentures retained by two or four implants are established treatment options that can improve prosthesis stability and function in edentulous patients [3,4].

The clinical performance of implant-supported overdentures is influenced by prosthesis retention, attachment design, maintenance requirements, and the biomechanical interaction between the prosthesis and supporting implants. Bar, ball, Locator-type, and other stud or resilient attachment systems differ in geometry, material composition, retentive mechanism, and functional behavior. Contemporary systematic evidence indicates high implant and overdenture survival across different attachment systems, while prosthetic maintenance and complication profiles vary among designs [5,6]. Long-term clinical studies also support the functional benefit of implant-retained mandibular overdentures and show that patient satisfaction, peri-implant outcomes, and maintenance requirements can vary with attachment strategy [7,8,9,10,11].

Overdenture attachments contain interacting metallic, polymeric, or combined retentive interfaces. Repeated insertion and removal may produce wear, deformation, and changes in frictional engagement, progressively altering retentive force [6]. Recent in vitro evidence has demonstrated attachment-specific retention loss and wear patterns for ball, Locator R-Tx, and other resilient attachment systems after simulated aging [12]. Earlier laboratory studies likewise demonstrated that wear behavior is influenced by attachment design, material pairing, lubrication, and repeated insertion–removal [13,14].

Attachment components are also exposed to water, temperature fluctuations, and chemical challenges in the oral environment. A systematic review showed that temperature, pH, cleansing media, and aging can alter attachment retention, with responses varying according to material and attachment configuration [15]. Therefore, mechanical aging and thermal cycling should be considered separately when characterizing the durability of retentive interfaces.

Polyurethane resin has been used as a standardized experimental substrate in implant biomechanical studies because of its relatively homogeneous mechanical properties and suitability for controlled laboratory testing [16,17,18]. Accordingly, a polyurethane mandibular model was selected to provide standardized testing conditions in the present study.

Although bar, ball, and Locator-type attachments have been extensively investigated, contemporary evidence for alternative snap-type retentive systems remains comparatively limited. Recent studies have expanded the evidence for snap-type and resilient attachment systems used in implant overdentures [19]. Nevertheless, evidence specifically addressing the aging behavior of TC-Snap remains limited. In addition, most retention studies emphasize axial or overall pull-off values, whereas the influence of force-application location and dislodgement geometry has received less attention. Characterizing both aging-related retention change and spatial variation in pull-off behavior may provide a more complete description of attachment performance.

A four-implant configuration was selected to provide a common experimental model accommodating both splinted bar and unsplinted attachment systems under the same implant distribution. Four-implant configurations have also been investigated in previous clinical studies of mandibular implant-supported overdentures [3,4]. This design allowed attachment-specific overdenture specimens to be evaluated while maintaining consistent implant positions, interimplant distances, and implant orientation. The evaluation of unilateral and bilateral force-application points was intended to characterize whether retentive behavior varied with the location and geometry of dislodgement, rather than to directly simulate masticatory loading. Therefore, the purpose of this descriptive pilot in vitro study was to characterize changes in retentive force in four attachment-specific overdenture specimens—distal extension bar, ball/O-ring, Locator R-Tx, and TC-Snap—following mechanical aging and thermal cycling, and to describe retention patterns under unilateral and bilateral force-application geometries.

## 2. Materials and Methods

### 2.1. Experimental Model and Implant Placement

**Figure 1:**
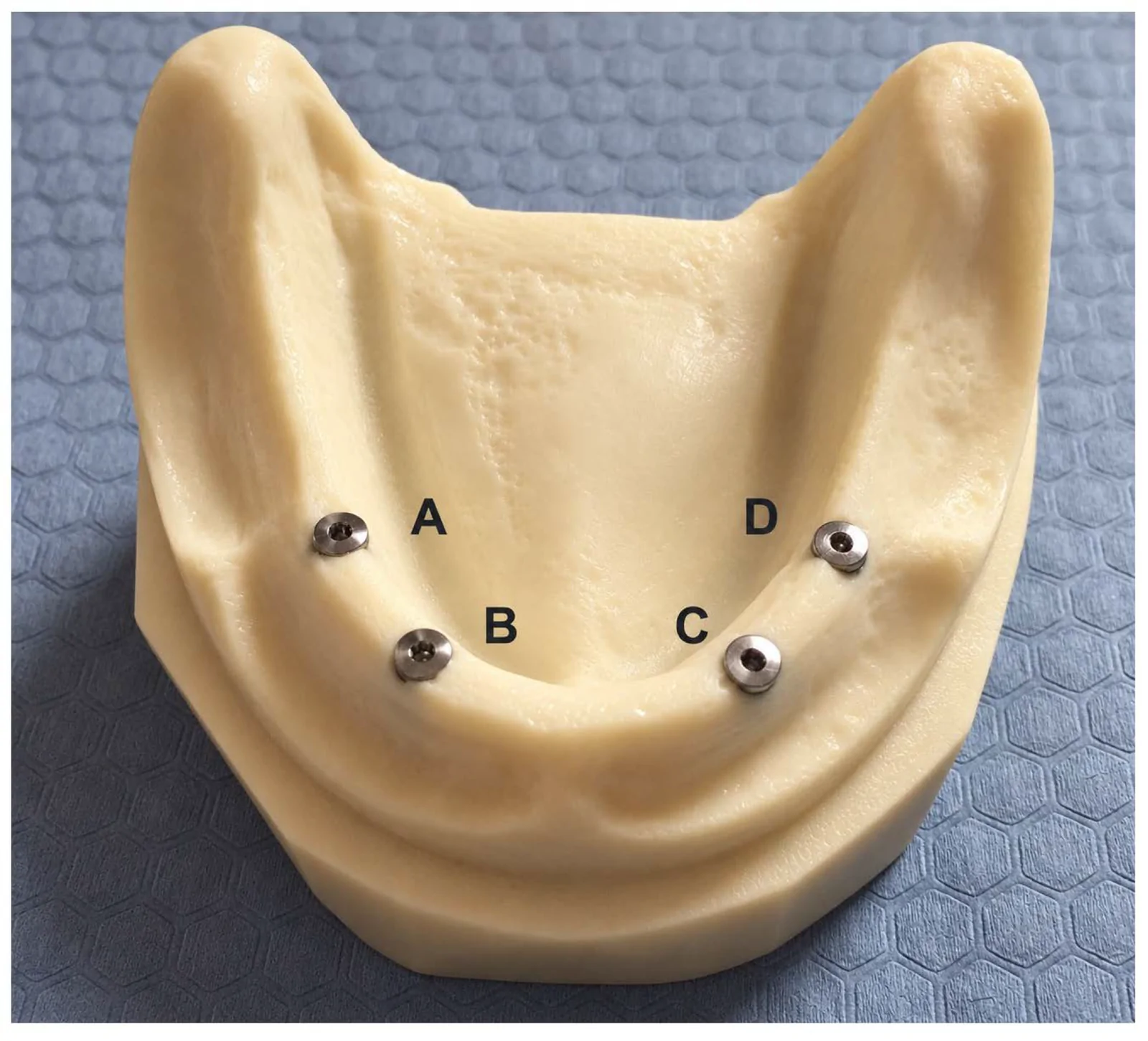
Edentulous mandibular polyurethane model with four implants positioned in the interforaminal region. Implant positions were designated as A, B, C, and D.

This descriptive pilot in vitro study evaluated the retentive force of four attachment-specific overdenture specimens under standardized mechanical and thermal aging conditions. A standardized edentulous mandibular model was fabricated by pouring fluid polyurethane resin (PU F16; Axson, Cergy, France), prepared according to the manufacturer’s instructions, into a silicone mold of an edentulous mandible. Four implant sockets, each 11.5 mm deep and spaced 11 mm apart center-to-center, were prepared manually according to the surgical drilling protocol for the 3.5 × 11.5 mm implants, with the sockets positioned as parallel to each other as possible. Four SLA-coated screw-type implants measuring 3.5 mm in diameter and 11.5 mm in length (Master Screw 3.5 × 11.5; Bredent Medical, Senden, Germany) were inserted into the prepared sockets according to the manufacturer’s instructions. The prosthetic components were connected to the implants and tightened to 20 N·cm using a mechanical torque wrench. The implant positions were designated A, B, C, and D in a counterclockwise direction (Figure 1). The same four implants embedded in the standardized mandibular model were retained throughout the study. For each attachment condition, the implant-level prosthetic components were completely replaced with those specific to the attachment system being evaluated, while implant position, interimplant distance, and implant orientation remained unchanged.

### 2.2. Attachment Systems and Overdenture Fabrication

**Figure 2:**
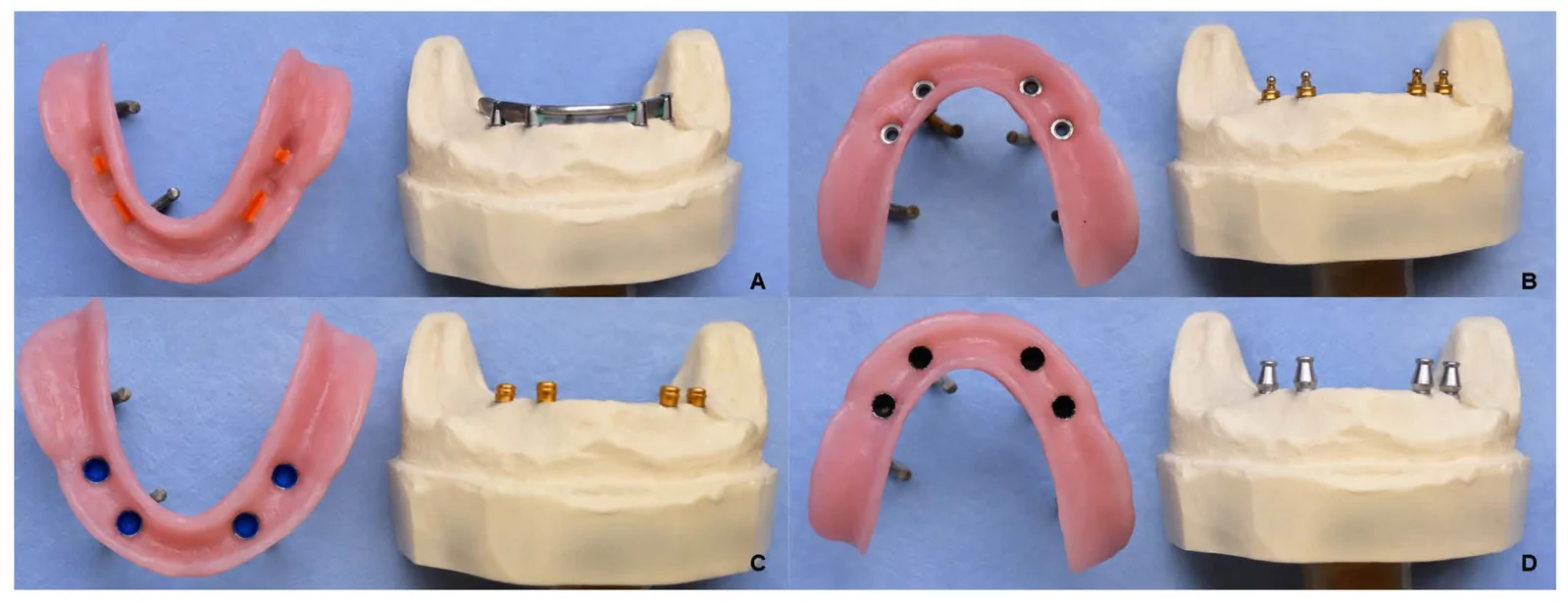
Overdenture bases and mandibular models prepared with the evaluated attachment systems: (**A**) distal extension bar attachment, (**B**) ball attachment, (**C**) Locator R-Tx attachment, and (**D**) TC-Snap attachment.

Four attachment systems were evaluated: distal extension bar, ball, Locator R-Tx, and TC-Snap attachments (Figure 2). For the distal extension bar group, a bar framework with a 10 mm distal cantilever was fabricated. A green plastic spacer was positioned on the bar framework to maintain the position of the retentive assembly during acrylic resin processing, and areas outside the spacer and retentive regions were blocked out with wax. For the ball attachment group, ball abutments were connected to the implants, and the corresponding O-ring housings (Bredent Medical, Senden, Germany) were seated over the abutments and incorporated into the overdenture base. For the Locator group, Locator R-Tx abutments (Zest Anchors LLC, Carlsbad, CA, USA) were connected to the implants, and the corresponding housings were incorporated into the overdenture base. For the TC-Snap group, primary crowns were connected to the implants, and the corresponding secondary TC-Snap components (TC-Snap-in part; Bredent Medical, Senden, Germany) were fabricated by laser sintering and incorporated into the overdenture base.

Wax patterns simulating mandibular overdenture bases were invested in conventional denture flasks. Following wax elimination, heat-polymerized polymethyl methacrylate (PMMA) resin (IQ-15; Imicryl, Konya, Türkiye) was processed using a short polymerization cycle at 100 °C for 20 min according to the manufacturer’s instructions. The processed overdenture bases were subsequently finished and polished. In the distal extension bar group, the spacer was removed, and the orange Hader retentive components were placed in the overdenture base. In the Locator R-Tx group, the black processing inserts were removed and replaced with blue retentive inserts. In the TC-Snap group, Retention.sil 600 (Bredent Medical, Senden, Germany) was applied according to the manufacturer’s instructions.

### 2.3. Retention Testing

Retention testing was performed using a universal testing machine (Instron 3344; Instron Corp., Canton, MA, USA). Four hook screws of identical size were positioned at equal distances on the external surface of each overdenture base and aligned parallel to one another using a dental surveyor. The hooks were connected to a four-chain pulling assembly attached to a 5 × 5 cm^2^ plate positioned 15 cm from the external surface of the overdenture.

Pull-out tests were performed at four unilateral test points corresponding to implant positions A, B, C, and D and at three bilateral test points (A–B, B–C, and C–D). Tensile force was applied through the corresponding chain configuration at a crosshead speed of 2 mm/min until complete disengagement of the overdenture from the attachment system, and the maximum force recorded during dislodgement was defined as the retentive force (N). Before each measurement, the overdenture was completely reseated on the corresponding attachment system.

A separate overdenture base and attachment-specific prosthetic components were used for each attachment condition. Only the four implants embedded in the polyurethane model were common to all experimental conditions. Implant positions A–D, interimplant distance, and implant orientation therefore remained constant across attachment conditions. Each overdenture base was used throughout the corresponding experimental procedures. At each test point and measurement interval, ten consecutive pull-out measurements were obtained from the same attachment-specific overdenture specimen. These measurements were considered technical replicates rather than independent experimental units, and their mean was calculated to obtain the retentive-force value for each specimen, test point, and measurement interval.

### 2.4. Mechanical Aging

Mechanical aging was performed manually by the same operator under dry laboratory conditions. No automated or mechanically guided device was used to standardize the insertion–removal trajectory, angulation, or seating force, and no separate assessment of procedural reproducibility was performed. Each insertion–removal cycle consisted of complete seating of the overdenture on the attachment components followed by complete removal. A total of 540 and 1080 insertion–removal cycles were performed. Based on an assumption of three insertion–removal events per day, these cumulative intervals represented approximately 6 and 12 months of simulated clinical use, respectively; this cycle-to-time relationship was treated as an experimental approximation because published aging protocols vary in their assumptions regarding daily prosthesis removal [6]. A single pre-aging baseline retentive-force measurement was obtained for each attachment-specific specimen and was used as the common reference for both the mechanical-aging and thermal-cycling evaluations. Retentive force was measured after 540 cumulative insertion–removal cycles. Mechanical aging was then continued using the same specimen for an additional 540 cycles, resulting in a total of 1080 cumulative cycles, after which retentive force was measured again.

### 2.5. Thermal Cycling

Thermal cycling was performed using a thermocycling unit (Gökçeler Makina, Sivas, Türkiye) between water baths maintained at 5 ± 1 °C and 55 ± 1 °C. The overdenture specimen was exposed to each water bath, with a dwell time of 30 s and a transfer time of 5 s between baths. The specimens were subjected to 10,000 thermal cycles, with retentive-force measurements performed after 5000 and 10,000 cycles and evaluated relative to the common pre-aging baseline described above. No direct clinical-time equivalence was assigned to the thermal-cycle counts [15].

### 2.6. Statistical and Descriptive Analysis

Descriptive analyses were performed using SigmaPlot software (version 12.3; Systat Software Inc., San Jose, CA, USA). For each attachment-specific overdenture specimen, test point, and measurement interval, ten consecutive pull-out measurements obtained from the same specimen were treated as technical replicates and summarized as mean and standard deviation (SD). Changes in retentive force following mechanical aging and thermal cycling were characterized using absolute retentive force (N), absolute change from baseline (N), percentage retention loss, and percentage retention preservation. Percentage retention loss was calculated as [(baseline retentive force − post-aging retentive force)/baseline retentive force] × 100, whereas retention preservation was calculated as (post-aging retentive force/baseline retentive force) × 100. Changes occurring during successive aging intervals were also evaluated descriptively to characterize the pattern of retention degradation over time. To describe the influence of force-application geometry, mean retentive forces obtained from unilateral test points (A, B, C, and D) and bilateral test points (A–B, B–C, and C–D) were summarized separately, and the bilateral-to-unilateral retention ratio was calculated. Final retentive-force values were additionally compared descriptively with retentive-force values previously proposed in the literature. These values were used solely for descriptive comparison and were not considered validated clinical thresholds for the present four-implant experimental configuration. Because only one attachment-specific overdenture specimen was fabricated for each condition, repeated measurements were not considered independent experimental units. Accordingly, inferential statistical comparisons and *p*-values were not used for comparisons among attachment conditions, test points, or aging intervals to avoid pseudoreplication.

## 3. Results

### 3.1. Mechanical Aging

Retentive-force values following mechanical aging are presented in Table 1. Retentive force decreased progressively in each of the four attachment-specific overdenture specimens. Based on descriptive averages across the seven force-application points, retention in the distal extension bar specimen decreased from 67.77 N at baseline to 63.24 N after 540 cycles and 45.75 N after 1080 cycles, corresponding to a final retention loss of 32.50%. The corresponding values decreased from 32.76 to 27.13 N (17.18% loss) in the ball/O-ring specimen, from 36.22 to 30.41 N (16.06% loss) in the Locator R-Tx specimen, and from 28.95 to 24.06 N (16.91% loss) in the TC-Snap specimen. Descriptively, the tested distal extension bar specimen had the highest absolute retentive-force values and the greatest proportional loss among the four specimens.

Point-specific retention loss after 1080 cycles ranged from 11.63% to 21.04% in the ball/O-ring specimen, 23.70% to 39.63% in the distal extension bar specimen, 10.21% to 21.59% in the Locator R-Tx specimen, and 11.86% to 21.20% in the TC-Snap specimen. Mean unilateral/bilateral losses were 15.28%/19.45% for the ball/O-ring specimen, 32.88%/31.98% for the distal extension bar specimen, 14.95%/17.29% for the Locator R-Tx specimen, and 15.60%/18.52% for the TC-Snap specimen. Retentive-force values at all seven force-application points in each of the four tested specimens remained above 20 N after 1080 cycles; this value is reported solely for descriptive comparison with values previously proposed in the literature [6].

### 3.2. Thermal Aging

Retentive-force values following thermal cycling are presented in Table 2. Based on descriptive averages across the seven force-application points, retention in the distal extension bar specimen decreased from 67.77 N at baseline to 47.74 N after 5000 cycles and 35.63 N after 10,000 cycles, corresponding to a final retention loss of 47.42%. The corresponding values decreased from 32.76 to 22.90 N (30.11% loss) in the ball/O-ring specimen, from 36.22 to 25.07 N (30.78% loss) in the Locator R-Tx specimen, and from 28.95 to 20.84 N (28.02% loss) in the TC-Snap specimen.

Point-specific retention loss after 10,000 thermal cycles ranged from 20.95% to 35.22% in the ball/O-ring specimen, 41.89% to 53.14% in the distal extension bar specimen, 20.76% to 42.58% in the Locator R-Tx specimen, and 22.22% to 31.93% in the TC-Snap specimen. Mean unilateral/bilateral losses were 28.57%/31.94% for the ball/O-ring specimen, 48.22%/46.33% for the distal extension bar specimen, 26.94%/35.04% for the Locator R-Tx specimen, and 27.37%/28.84% for the TC-Snap specimen. After 10,000 cycles, retentive-force values at all seven points remained above 20 N in the tested distal extension bar and Locator R-Tx specimens; six of seven points in the ball/O-ring specimen and five of seven points in the TC-Snap specimen remained above this value. These observations are descriptive and do not imply a validated clinical threshold for the present four-implant configuration [6].

### 3.3. Retentive-Force Patterns According to Force-Application Geometry

At baseline, mean bilateral retentive force was greater than mean unilateral retentive force in the tested ball/O-ring (34.88 vs. 31.18 N), Locator R-Tx (40.14 vs. 33.29 N), and TC-Snap (30.22 vs. 28.00 N) specimens, whereas similar bilateral and unilateral values were observed in the distal extension bar specimen (66.74 vs. 68.55 N). The corresponding baseline bilateral-to-unilateral ratios were 1.12, 1.21, 1.08, and 0.97, respectively.

To facilitate interpretation of force-application geometry, bilateral-to-unilateral retention ratios were calculated, with a value of 1.00 indicating similar mean retention under the two dislodgement conditions, values above 1.00 indicating greater bilateral retention, and values below 1.00 indicating greater unilateral retention. After 1080 insertion–removal cycles, these ratios were 1.06 for the ball/O-ring specimen, 0.99 for the distal extension bar specimen, 1.17 for the Locator R-Tx specimen, and 1.04 for the TC-Snap specimen. After 10,000 thermal cycles, the corresponding ratios were 1.07, 1.01, 1.07, and 1.06. Descriptively, the tested distal extension bar specimen showed nearly equivalent unilateral and bilateral mean retention, whereas somewhat higher bilateral than unilateral values were observed in the three tested unsplinted specimens. These observations are descriptive and do not represent inferential comparisons among attachment systems or force-application geometries.

## 4. Discussion

The present descriptive pilot in vitro study characterized the retention behavior of four attachment-specific overdenture specimens following mechanical and thermal aging and across different force-application geometries. Three principal observations emerged from the tested specimens. First, retentive force generally decreased with aging in each specimen, although the magnitude and point-specific pattern of loss varied. Second, the tested distal extension bar specimen exhibited the highest absolute retentive-force values throughout both aging protocols and also showed the greatest proportional retention loss, reaching 32.50% after 1080 insertion–removal cycles and 47.42% after 10,000 thermal cycles. Third, point-specific and unilateral–bilateral retention patterns varied among the four tested specimens. Taken together, these descriptive observations indicate that, within the present experimental model, absolute retentive force, proportional retention loss during aging, and dislodgement geometry each provided distinct information about the behavior of the tested specimens. Because only one attachment-specific specimen was evaluated for each attachment condition, these observations should not be interpreted as system-level estimates or inferential comparisons among attachment systems.

In the tested distal extension bar specimen, high absolute retentive-force values were accompanied by a relatively large proportional retention loss during aging. Denewar et al. [20] reported that a cantilevered Hader bar retained by three plastic clips produced high retention while also exhibiting substantial retention loss after repeated insertion and withdrawal, a pattern broadly consistent with the observation in the present bar specimen. Earlier fatigue testing of Locator attachments and Hader clips also documented time-dependent changes in retention [21]. Progressive alteration of the clip-bar interface through wear, deformation, or changes in frictional engagement may provide a possible explanation for such changes. Recent clinical studies have quantified wear of nylon clips and have directly compared retention behavior using different clip materials [22,23]. However, the proportional retention loss observed in the distal extension bar specimen in the present study should not be generalized to bar attachments as a whole, because retention changes may depend on bar geometry, cantilever configuration, clip number and material, initial engagement, and aging conditions. Because wear and deformation were not measured in the present study, these mechanisms remain literature-supported possible explanations rather than directly demonstrated causes. Earlier comparative investigations of bar, stud, and resilient attachments have also reported differences in initial retention and fatigue behavior among attachment configurations [24,25,26,27].

In the tested Locator R-Tx specimen, proportional retention loss was 16.06% after mechanical aging and 30.78% after thermal cycling. Previous studies have also reported changes in Locator R-Tx retention during aging. Abdelaziz et al. [28] reported a 26.31% retention loss after 5475 cycles using independently replicated overdentures, whereas Wakam et al. [12] observed substantial retention loss and wear after 10,000 insertion–removal cycles. Quantitative and qualitative analyses have also demonstrated wear-related changes in Locator R-Tx retentive components [29]. Kamonkhantikul et al. [30] reported changes in Locator R-Tx retention after aqueous storage and found that subsequent thermocycling did not produce a uniform progressive reduction across insert configurations; they also demonstrated material-dependent water absorption. Although these findings provide possible explanations for aging-related changes in Locator R-Tx retention, water absorption, wear, and material degradation were not evaluated in the present study. Therefore, the 30.78% retention loss observed in the tested Locator R-Tx specimen after thermal cycling should be interpreted as a descriptive change under the applied thermal-aging protocol and should not be attributed to a specific degradation mechanism. Previous Locator-focused studies have further shown that retentive force may be influenced by insert/attachment design, implant angulation, and repeated use [31,32].

In the tested ball/O-ring specimen, proportional retention loss was 17.18% after mechanical aging and 30.11% after thermal cycling. Wakam et al. [12] demonstrated attachment-specific wear at interacting ball-system surfaces during cyclic aging. A systematic review focused on ball attachments and O-rings likewise concluded that retention generally decreases with increasing insertion–removal cycles, while the magnitude of loss depends on O-ring material and dimensions, ball characteristics, implant angulation, and the fatigue protocol [33]. Abdelaziz et al. [28] reported a 19.87% retention loss for a ball-and-socket attachment after a longer fatigue protocol. Because material changes in the O-ring were not directly evaluated in the present study, the observed retention changes cannot be attributed to a specific wear or degradation mechanism. Earlier ball/O-ring studies similarly demonstrated that both attachment design and non-axial dislodgement can alter measured retention [34,35].

In the tested TC-Snap specimen, proportional retention loss was 16.91% after mechanical aging and 28.02% after thermal cycling. Contemporary evidence specifically addressing TC-Snap aging remains limited. Mourad et al. [19] reported progressive retention loss for a related Ti-Si Snap system incorporating Retention.sil; however, that attachment differs from the TC-Snap–Retention.sil 600 configuration evaluated in the present study. Recent thermomechanical studies of other resilient attachment systems have also reported retention changes during aging, although these findings cannot be directly extrapolated to TC-Snap [36]. The retention changes observed in the present TC-Snap specimen should therefore be interpreted within the specific experimental configuration and aging protocols used in this study. Polyvinyl siloxane matrix materials are themselves retention-level dependent; Retention.sil 600 has been reported to generate higher retention than lower-retention PVS formulations [37].

Mechanical aging was associated with changes in retention in each of the four tested specimens, with variation in the magnitude and spatial pattern of these changes. Systematic evidence shows substantial heterogeneity among insertion–removal protocols and indicates that retention degradation may be influenced by attachment geometry, retentive material, initial engagement, dislodgement rate, and aging conditions [6]. The 540- and 1080-cycle intervals used in the present study, based on three daily insertion–removal events, should therefore be interpreted as standardized experimental approximations rather than exact clinical-time equivalents. Mechanical aging was performed manually by a single operator under dry laboratory conditions. Mechanical aging was performed manually by the same operator throughout the protocol. Although this approach provided operator consistency, the insertion–removal procedure was not mechanically controlled; therefore, some operator-dependent variability cannot be excluded. The retention-loss patterns observed in the four specimens therefore characterize their behavior under the specific mechanical-aging protocol used in this study. A recent comparison using 270, 540, and 1080 insertion–removal cycles reported that a Hader bar maintained high absolute retentive-force values throughout aging [38].

Different numerical patterns of retention change were observed in the four tested specimens after thermal cycling. Previous evidence indicates that environmental factors such as temperature, water exposure, pH, and cleansing solutions may influence attachment retention in a material-dependent manner [15]. Studies of Locator, Locator R-Tx, and Novaloc inserts have further shown that water storage and thermocycling can contribute differently to retention change and water uptake [30]. Shastry et al. [24] reported reduced retentive force after thermocycling in ball/O-ring, Hader bar/clip, and Locator systems, while more recent experimental work has combined thermal cycling with an acidic aqueous challenge and subsequent insertion–removal cycling in Locator, Locator R-Tx, and OT Equator inserts [39]. The numerically greater percentage losses after 10,000 thermal cycles than after 1080 insertion–removal cycles should not be interpreted as indicating that thermal aging was intrinsically more damaging, because the two protocols represented different exposure modalities and magnitudes and were not designed as equivalent aging doses.

Retention loss was not spatially uniform across the tested force-application points. After mechanical aging, point-specific losses ranged from 10.21% to 39.63%, whereas after thermal aging they ranged from 20.76% to 53.14% across the tested specimens and force-application points. Analysis of the force-application points also revealed different unilateral and bilateral retention patterns. Contemporary biomechanical evidence supports the importance of force location and distribution. Petricevic et al. [40] demonstrated that loading position, implant number, force magnitude, and splinting modify strain patterns in mandibular overdentures, while Gumprecht et al. [41] showed that pull-off force varies with attachment system, implant configuration, and axial versus non-axial dislodgement. In the present experiment, the tested distal extension bar specimen showed similar mean retention under unilateral and bilateral dislodgement, with bilateral-to-unilateral ratios remaining close to 1.0, whereas the three tested unsplinted specimens generally showed somewhat higher retention under bilateral dislodgement. These differences indicate that the location and geometry of the applied dislodging force were associated with different descriptive retention patterns in the tested specimens. However, prosthesis rotation, attachment-level load sharing, sequence of attachment disengagement, three-dimensional displacement, and strain were not directly measured; therefore, the biomechanical mechanisms underlying these patterns cannot be determined from the present experiment. Importantly, the unilateral and bilateral pull-out conditions used here represent controlled dislodgement geometries and should not be interpreted as direct simulations of unilateral or bilateral mastication. Earlier studies have also demonstrated effects of implant number, distribution, interimplant distance, and attachment type on overdenture retention and stability [42,43,44].

Clinical interpretation requires consideration of both proportional retention loss and residual absolute retentive force. Values of approximately 5–20 N have been proposed in the literature for different implant-overdenture configurations, but these values are not universally validated clinical thresholds [6]. In the present four-implant model, the approximately 20-N value was therefore used solely as a descriptive reference. Retentive-force values at all force-application points remained above 20 N after mechanical aging. After thermal aging, values remained above 20 N at all seven points in the distal extension bar and Locator R-Tx specimens, at six of seven points in the ball/O-ring specimen, and at five of seven points in the TC-Snap specimen. Values below 20 N should not be classified as clinically inadequate on the basis of the present experiment. Clinical attachment selection also depends on factors beyond retentive force, including maintenance requirements, wear, prosthetic complications, and patient-reported outcomes [5,6]. Accordingly, the retention values observed in the present specimens should be interpreted descriptively and should not be used alone to establish clinical superiority among attachment systems. Long-term clinical and maintenance literature further indicates that patient satisfaction and prosthetic maintenance cannot be inferred from pull-off force alone [45].

Several limitations should be considered. Only the four implants embedded in the standardized mandibular model were common to all attachment conditions; the implant-level prosthetic components and overdenture base were attachment-specific and were completely replaced for each condition. This design maintained the same implant distribution, interimplant distance, and implant orientation across attachment conditions. Although the implant sockets were prepared parallel to each other, no separate quantitative or radiographic verification of implant parallelism was documented. However, only one attachment-specific overdenture specimen was fabricated for each condition. Consequently, assembly-specific variation related to factors such as overdenture-base adaptation, component positioning, processing procedures, and insertion–removal handling may have contributed to the observed retention values. The influence of these factors cannot be separated from that of attachment configuration in the present design. The ten consecutive pull-out measurements at each point and interval were technical replicates from the same specimen rather than independent experimental units; they characterize within-specimen measurement variability but do not provide independent replication at the attachment-system level. Consequently, the findings are descriptive and hypothesis-generating, and inferential *p*-value-based comparisons were not used to avoid pseudoreplication. The standardized polyurethane model did not reproduce resilient mucosal support, saliva, patient-specific anatomy, neuromuscular control, or dynamic occlusal function. Only one four-implant distribution and one configuration of each attachment were evaluated. Mechanical and thermal aging were performed separately, whereas clinical attachments are exposed to interacting mechanical, thermal, and chemical challenges in the oral environment. Mechanical aging was performed manually under dry laboratory conditions by the same operator throughout the aging protocol. Mechanical aging was performed manually by the same operator throughout the protocol. Although this approach provided operator consistency, the insertion–removal procedure was not mechanically controlled; therefore, some operator-dependent variability cannot be excluded. Because a single pre-aging baseline measurement was used as the common reference for both aging protocols, protocol-specific baseline variability could not be independently characterized. Material and surface changes associated with aging were not directly characterized. The exact direction and angulation of the applied dislodging force at each force-application point were not independently quantified. Finally, point-specific pull-out tests represented controlled dislodgement geometries rather than direct simulations of mastication. Future studies should use independently replicated attachment-specific overdenture specimens, combined thermomechanical aging, multidirectional retention testing, three-dimensional displacement or strain analysis, and quantitative surface and material characterization to establish reproducible structure–wear–retention relationships.

## 5. Conclusions

Within the limitations of this descriptive pilot in vitro study, retentive force decreased after mechanical and thermal aging in each of the four attachment-specific overdenture specimens, although the magnitude and point-specific pattern of retention loss varied. The tested distal extension bar specimen showed the highest absolute retentive-force values throughout both aging protocols and also exhibited the greatest proportional retention loss. The tested ball/O-ring, Locator R-Tx, and TC-Snap specimens showed lower absolute retentive-force values and smaller proportional reductions under the experimental conditions used. These descriptive observations indicate that absolute retentive force and proportional retention loss during aging represent distinct aspects of the retention behavior observed in the tested specimens.

Retentive-force values also varied according to the location and distribution of the applied dislodging force. Point-specific retention loss was not uniform, and unilateral and bilateral force-application geometries produced different descriptive patterns across the tested specimens and aging conditions. These observations suggest that both the magnitude and location of the applied dislodging force are relevant when characterizing retention behavior under the present experimental conditions.

These findings should be interpreted within the descriptive pilot nature of the study. Further studies with independent experimental replication and clinically relevant aging conditions are needed to determine the reproducibility and clinical relevance of the observed attachment-specific retention patterns.

## Figures and Tables

**Table 1:** Retentive-force values and percentage retention loss after mechanical aging according to attachment system and force-application point.

Attachment System	Force-Application Point	Baseline, N Mean ± SD	540 Cycles, N Mean ± SD	Loss at 540 Cycles (%)	1080 Cycles, N Mean ± SD	Loss at 1080 Cycles (%)
Ball	A	28.84 ± 0.80	27.23 ± 0.48	5.58	24.64 ± 0.69	14.56
Ball	B	30.69 ± 0.97	29.45 ± 0.66	4.04	27.12 ± 0.64	11.63
Ball	C	33.12 ± 1.33	31.29 ± 0.44	5.53	27.65 ± 0.53	16.52
Ball	D	32.05 ± 0.18	29.41 ± 0.36	8.24	26.24 ± 0.60	18.13
Ball	A–B	33.13 ± 0.49	29.59 ± 0.48	10.69	26.16 ± 0.50	21.04
Ball	B–C	36.35 ± 0.50	32.23 ± 0.51	11.33	30.03 ± 0.53	17.39
Ball	C–D	35.15 ± 0.65	31.33 ± 0.33	10.87	28.09 ± 0.56	20.09
Ball	Unilateral mean	31.18	29.34	5.87	26.41	15.28
Ball	Bilateral mean	34.88	31.05	10.97	28.09	19.45
Bar	A	55.29 ± 1.00	54.32 ± 1.96	1.75	35.84 ± 0.65	35.18
Bar	B	77.05 ± 1.68	72.98 ± 0.85	5.28	55.30 ± 0.50	28.23
Bar	C	86.45 ± 1.79	72.47 ± 0.64	16.17	56.39 ± 0.66	34.77
Bar	D	55.40 ± 1.03	53.14 ± 1.00	4.08	36.51 ± 0.59	34.10
Bar	A–B	60.72 ± 0.45	55.31 ± 0.88	8.91	42.64 ± 0.28	29.78
Bar	B–C	80.89 ± 0.65	77.82 ± 1.46	3.80	48.83 ± 0.38	39.63
Bar	C–D	58.61 ± 0.88	56.65 ± 1.80	3.34	44.72 ± 0.60	23.70
Bar	Unilateral mean	68.55	63.23	7.76	46.01	32.88
Bar	Bilateral mean	66.74	63.26	5.21	45.40	31.98
Locator R-Tx	A	31.72 ± 1.20	30.78 ± 0.62	2.96	26.72 ± 0.40	15.76
Locator R-Tx	B	32.33 ± 0.64	31.86 ± 0.58	1.45	29.03 ± 0.55	10.21
Locator R-Tx	C	36.18 ± 0.27	33.45 ± 0.34	7.55	29.63 ± 0.52	18.10
Locator R-Tx	D	32.92 ± 0.33	31.37 ± 0.47	4.71	27.86 ± 0.63	15.37
Locator R-Tx	A–B	35.15 ± 0.43	34.52 ± 0.30	1.79	31.19 ± 0.38	11.27
Locator R-Tx	B–C	42.62 ± 0.99	38.49 ± 0.18	9.69	34.97 ± 0.56	17.95
Locator R-Tx	C–D	42.65 ± 0.59	36.64 ± 0.23	14.09	33.44 ± 0.50	21.59
Locator R-Tx	Unilateral mean	33.29	31.87	4.27	28.31	14.95
Locator R-Tx	Bilateral mean	40.14	36.55	8.94	33.20	17.29
TC-Snap	A	26.94 ± 1.22	24.53 ± 0.48	8.95	22.24 ± 0.43	17.45
TC-Snap	B	29.34 ± 0.68	26.61 ± 0.35	9.30	24.39 ± 1.27	16.87
TC-Snap	C	28.49 ± 1.08	27.36 ± 0.43	3.97	25.11 ± 0.64	11.86
TC-Snap	D	27.23 ± 0.49	25.21 ± 0.38	7.42	22.79 ± 0.50	16.31
TC-Snap	A–B	28.65 ± 0.82	26.11 ± 0.55	8.87	24.23 ± 0.58	15.43
TC-Snap	B–C	32.07 ± 0.50	26.87 ± 0.22	16.21	25.27 ± 0.54	21.20
TC-Snap	C–D	29.93 ± 0.27	26.11 ± 0.56	12.76	24.36 ± 0.38	18.61
TC-Snap	Unilateral mean	28.00	25.93	7.40	23.63	15.60
TC-Snap	Bilateral mean	30.22	26.36	12.75	24.62	18.52

Values at individual force-application points are the mean ± SD of 10 consecutive technical pull-out measurements obtained from the same attachment-specific overdenture specimen. Retention loss (%) was calculated relative to the corresponding baseline value. Unilateral and bilateral summary rows are descriptive averages of A–D and A–B/B–C/C–D points, respectively, and are not means of independent experimental specimens.

**Table 2:** Retentive-force values and percentage retention loss after thermal aging according to attachment system and force-application point.

Attachment System	Force-Application Point	Baseline, N Mean ± SD	5000 Cycles, N Mean ± SD	Loss at 5000 Cycles (%)	10,000 Cycles, N Mean ± SD	Loss at 10,000 Cycles (%)
Ball	A	28.84 ± 0.80	25.99 ± 0.42	9.88	19.60 ± 0.42	32.04
Ball	B	30.69 ± 0.97	27.76 ± 0.35	9.55	24.26 ± 0.35	20.95
Ball	C	33.12 ± 1.33	27.76 ± 0.29	16.18	24.43 ± 0.29	26.24
Ball	D	32.05 ± 0.18	27.46 ± 0.32	14.32	20.78 ± 0.32	35.16
Ball	A–B	33.13 ± 0.49	26.65 ± 0.30	19.56	23.13 ± 0.30	30.18
Ball	B–C	36.35 ± 0.50	30.21 ± 0.32	16.89	25.31 ± 0.32	30.37
Ball	C–D	35.15 ± 0.65	28.38 ± 0.41	19.26	22.77 ± 0.41	35.22
Ball	Unilateral mean	31.18	27.24	12.61	22.27	28.57
Ball	Bilateral mean	34.88	28.41	18.53	23.74	31.94
Bar	A	55.29 ± 1.00	37.64 ± 0.42	31.92	28.88 ± 0.42	47.77
Bar	B	77.05 ± 1.68	59.24 ± 0.35	23.11	41.50 ± 0.35	46.14
Bar	C	86.45 ± 1.79	60.46 ± 0.29	30.06	40.51 ± 0.29	53.14
Bar	D	55.40 ± 1.03	38.43 ± 0.32	30.63	31.08 ± 0.32	43.90
Bar	A–B	60.72 ± 0.45	43.10 ± 0.30	29.02	34.01 ± 0.30	43.99
Bar	B–C	80.89 ± 0.65	49.43 ± 0.32	38.89	39.39 ± 0.32	51.30
Bar	C–D	58.61 ± 0.88	45.89 ± 0.41	21.70	34.06 ± 0.41	41.89
Bar	Unilateral mean	68.55	48.94	28.60	35.49	48.22
Bar	Bilateral mean	66.74	46.14	30.87	35.82	46.33
Locator R-Tx	A	31.72 ± 1.20	27.65 ± 0.42	12.83	22.62 ± 0.42	28.69
Locator R-Tx	B	32.33 ± 0.64	29.35 ± 0.35	9.22	25.62 ± 0.35	20.75
Locator R-Tx	C	36.18 ± 0.27	29.86 ± 0.29	17.47	25.57 ± 0.29	29.33
Locator R-Tx	D	32.92 ± 0.33	28.53 ± 0.32	13.34	23.47 ± 0.32	28.71
Locator R-Tx	A–B	35.15 ± 0.43	32.54 ± 0.30	7.43	25.14 ± 0.30	28.48
Locator R-Tx	B–C	42.62 ± 0.99	35.93 ± 0.32	15.70	28.60 ± 0.32	32.90
Locator R-Tx	C–D	42.65 ± 0.59	35.13 ± 0.41	17.63	24.49 ± 0.41	42.58
Locator R-Tx	Unilateral mean	33.29	28.85	13.34	24.32	26.94
Locator R-Tx	Bilateral mean	40.14	34.53	13.97	26.08	35.04
TC-Snap	A	26.94 ± 1.22	23.21 ± 0.42	13.85	18.63 ± 0.42	30.85
TC-Snap	B	29.34 ± 0.68	27.22 ± 0.35	7.23	21.76 ± 0.35	25.84
TC-Snap	C	28.49 ± 1.08	26.58 ± 0.29	6.70	22.16 ± 0.29	22.22
TC-Snap	D	27.23 ± 0.49	23.45 ± 0.32	13.88	18.80 ± 0.32	30.96
TC-Snap	A–B	28.65 ± 0.82	25.46 ± 0.30	11.13	21.27 ± 0.30	25.76
TC-Snap	B–C	32.07 ± 0.50	25.75 ± 0.32	19.71	21.83 ± 0.32	31.93
TC-Snap	C–D	29.93 ± 0.27	24.82 ± 0.41	17.07	21.41 ± 0.41	28.47
TC-Snap	Unilateral mean	28.00	25.11	10.30	20.34	27.37
TC-Snap	Bilateral mean	30.22	25.34	16.13	21.50	28.84

Values at individual force-application points are the mean ± SD of 10 consecutive technical pull-out measurements obtained from the same attachment-specific overdenture specimen. Retention loss (%) was calculated relative to the corresponding baseline value. Unilateral and bilateral summary rows are descriptive averages of A–D and A–B/B–C/C–D points, respectively, and are not means of independent experimental specimens.

## Data Availability

The original contributions presented in the study are included in the article. Further inquiries can be directed to the corresponding author.

## Abbreviations

PMMA	polymethyl methacrylate
PVS	polyvinyl siloxane
SD	standard deviation
SLA	sandblasted, large-grit, acid-etched
N	Newton
